# Boundary Criterion Validation for Predicting Clinical DIC During Delivery in Fibrinogen–FDP Plane Using Severe Placental Abruption, and Characteristics of Clinical DIC Coagulation–Fibrinolytic Activation

**DOI:** 10.3390/jcm14155179

**Published:** 2025-07-22

**Authors:** Katsuhiko Tada, Yasunari Miyagi, Ichiro Yasuhi, Keisuke Tsumura, Ikuko Emoto, Maiko Sagawa, Norifumi Tanaka, Kyohei Yamaguchi, Kazuhisa Maeda, Kosuke Kawakami

**Affiliations:** 1 Department of Obstetrics and Gynecology, National Hospital Organization, Okayama Medical Center, 1711-1 Tamasu, Kita Ward, Okayama 701-1192, Japan; 2 Medical Data Labo, 289-48 Yamasaki, Naka Ward, Okayama 703-8267, Japan; ymiyagi@mac.com; 3 Miyake Ofuku Clinic, 393-1 Ofuku, Minami Ward, Okayama 701-0204, Japan; 4 Department of Obstetrics and Gynecology, National Hospital Organization, Nagasaki Medical Center, 2-1001-1 Kubara, Omura 856-0835, Japan; iyasuhi0414@gmail.com; 5 Department of Obstetrics and Gynecology, National Hospital Organization, Saga Hospital, 1-20-1 Hinode, Saga 849-8577, Japan; tmrksk.obgy@gmail.com; 6 Department of Obstetrics and Gynecology, National Hospital Organization, Kyoto Medical Center, 1-1 Fukasakumukaihata, Kyoto 612-8555, Japan; ikuko_88@hotmail.com; 7 Department of Obstetrics and Gynecology, National Hospital Organization, Kure Medical Center, 3-1 Aoyama, Kure 737-0023, Japan; sagawa.maiko.ea@mail.hosp.go.jp; 8 Department of Obstetrics and Gynecology, National Hospital Organization, Higashihiroshima Medical Center, 513 Saijochojike, Higashihiroshima 739-0041, Japan; takyoubun@gmail.com; 9 Department of Obstetrics and Gynecology, National Hospital Organization, Mie Chuo Medical Center, 2158-5 Hisaimyojincho, Tsu 514-1101, Japan; kyokyokyo330@gmail.com; 10 Department of Obstetrics and Gynecology, National Hospital Organization, Shikoku Medical Center for Children and Adults, 2-1-1 Senyucho, Zentsuji 765-8507, Japan; maeda3981@gmail.com; 11 Department of Obstetrics and Gynecology, National Hospital Organization, Kokura Medical Center, 10-1 Harugaoka, Kokuraminami Ward, Kitakyushu 802-8533, Japan; kawakami.kosuke.ed@mail.hosp.go.jp

**Keywords:** disseminated intravascular coagulation, fibrinogen, fibrin/fibrinogen degradation products, macroscopic hematuria, placental abruption, plasmin-α_2_–plasmin inhibitor complex, postpartum hemorrhage, thrombin–antithrombin complex

## Abstract

**Background/Objectives:** We define severe postpartum hemorrhage (PPH) with macroscopic hematuria as clinical disseminated intravascular coagulation (DIC), a life-threatening condition. We also report a methodology using machine learning, a subtype of artificial intelligence, for developing the boundary criterion for predicting hematuria on the fibrinogen–fibrin/fibrinogen degradation product (FDP) plane. A positive FDP–fibrinogen/3–60 (mg/dL) value indicates hematuria; otherwise, non-hematuria is observed. We aimed to validate this criterion using severe placental abruption (PA), and to examine the activation of the coagulation–fibrinolytic system in clinical DIC. **Methods:** Of 17,285 deliveries across nine perinatal centers in Japan between 2020 and 2024, 13 had severe PA without hematuria, 18 had severe PPH without hematuria, and 3 had severe PPH with hematuria, i.e., clinical DIC. We calculated the values of the criterion formula for 13 cases of severe PA to validate the boundary criterion and compared the laboratory tests for coagulation–fibrinolytic activation among the three groups. **Results:** The calculated values using the criterion for the 13 PA without hematuria ranged from −108.91 to −5.87 and all were negative. In cases of clinical DIC, fibrinogen levels (median, 62 mg/dL) were lower (*p* < 0.05), while levels of FDP (96 mg/dL), the thrombin–antithrombin complex (120 ng/mL), and the plasmin-α_2_–plasmin inhibitor complex (28.4 μg/mL) were significantly higher than in the other two groups. **Conclusions:** This study demonstrated the validity of the boundary criterion for predicting hematuria using severe PA. The coagulation–fibrinolytic test results suggested that PPH cases with hematuria were assumed to have clinical DIC, indicating that this criterion may be considered for diagnosing DIC during delivery. However, further additional patient data are needed to confirm the usefulness of this criterion because of the very low number of hematuria cases.

## 1. Introduction

Disseminated intravascular coagulation (DIC) is a life-threatening condition, which means that an appropriate diagnosis is important for providing early treatment. Thus, proper diagnostic criteria are essential; three of these have been established recently for women who are not pregnant [1,2,3]. Enhanced coagulation activation is a major pathophysiology in DIC; however, the degree of fibrinolytic activation differs depending on the underlying disease, which is an important factor in characterizing DIC [4]. Obstetrical DIC, one of many critical conditions in obstetrics [5], is caused by placental abruption (PA), amniotic fluid embolism (AFE), and postpartum hemorrhage (PPH) [4,6,7,8]. It is classified in enhanced-fibrinolytic-type DIC characterized by marked fibrinolytic activation, leading fibrinogen decrease and FDP increase in laboratory tests [4,9]. Clinically, we must be aware that some findings may signify coagulopathy [10]; as such, we focused on hematuria seen in human [10,11] and animal models [4]. Additionally, if severe enough, DIC can cause organ dysfunction [1], and cases of PPH with hematuria could be assumed to be organ dysfunction [12]. Considering the above, the occurrence of hematuria associated with PPH as clinical DIC must be defined.

In the management of PPH, point-of-care testing has gained attention as a method of rapidly evaluating coagulopathy [13]. Two clinical diagnostic criteria were recently discovered for diagnosing obstetrical DIC: the pregnancy-modified ISTH DIC score [14] and new Japanese diagnostic criteria for obstetrical DIC [15]. However, an international consensus has yet to be reached [8]. In our previous study, among 13,368 total deliveries, 23 cases of severe PPH were observed, including 3 patients with hematuria [12]. In those patients with hematuria, fibrinogen was greatly decreased and FDP greatly increased compared with non-hematuria cases, indicating that cases with hematuria might have clinical DIC. Miyagi et al. [12] used artificial intelligence to develop a boundary formula for predicting hematuria on the fibrinogen–FDP plane: only a positive FDP–fibrinogen/3–60 (mg/dL) value indicates hematuria. This formula can also be considered as a criterion for predicting clinical DIC.

PA is a typical cause of obstetrical DIC and is thought to lead to DIC when severe [10,16]. Although the cohort data used to develop the boundary criterion [12] included a considerable number of PA cases, the criterion’s feasibility in PA cases remains unconfirmed. In this study, we hypothesized that the calculated values of the criterion formula would be positive in patients with hematuria and negative in patients without hematuria and aimed to validate the criterion using severe cases of PA. Then, we compared the laboratory tests values for coagulation–fibrinolytic activation in cases of clinical DIC, severe PA and severe PPH to clarify the characteristics of the coagulation–fibrinolytic profile in clinical DIC and other severe conditions during delivery.

## 2. Materials and Methods

### 2.1. Design and Study Population

This was a multicenter prospective case–series study of women who gave birth at any of the nine National Hospital Organization (NHO) perinatal centers in Japan between August 2020 and June 2024. The inclusion criteria were as follows: (1) >2000 mL of blood loss within 24 h of delivery, except for PA; (2) a clinical diagnosis of PA irrespective of blood loss; (3) any mode of delivery; (4) singleton or multiple pregnancies; and (5) delivery after 22 weeks of gestation. The exclusion criteria included medical complications that could cause coagulopathy and medications that affect the coagulation system, such as aspirin.

We developed a criterion for predicting hematuria using PPH cases with fibrinogen < 170 mg/dL [12], which is the initiation criterion for coagulation failure [17]. Therefore, we defined patients with fibrinogen < 170 mg/dL as having a severe form of the condition, and severe PPH and PA cases were also included. We then classified the included PPH and PA cases into the following three groups with or without hematuria: PPH with hematuria, PPH without hematuria, and PA without hematuria.

### 2.2. Clinical Data and Laboratory Tests

During vaginal delivery, blood loss was weighed using a blood-soaked pad and/or from direct blood collection using a collector bag. During cesarean delivery, blood-soaked surgical pads were weighed and/or blood volume aspirated from the surgical field was determined in milliliters. Blood loss was standardized in milliliters (mL). Obstetrical management and blood product transfusion were performed at the discretion of the attending physicians at each center; in this study, hematuria was defined via a physician’s diagnosis of macroscopic hematuria (MH). In cases with a diagnosis of MH, we confirmed that the patients did not have a urinary tract infection, urolithiasis, or any renal disease such as nephritis.

The attending physicians obtained blood samples when deemed clinically necessary, regardless of blood loss. Hemoglobin, platelet count, the prothrombin time–international normalized ratio, fibrinogen, FDP, and D-dimer were measured in each center’s laboratory for clinical management. Marked coagulation–fibrinolytic activation is the main pathophysiology of DIC in obstetrics [4]. Therefore, in this study, to accurately evaluate the coagulation–fibrinolytic activation in the patients, we measured the levels of the thrombin–antithrombin complex (TAT) and plasmin-α2–plasmin inhibitor complex (PIC), sensitive molecular markers of coagulation and fibrinolysis activation [18], respectively. TAT and PIC were measured using a chemiluminescent enzyme immunoassay and latex photometric immunoassay, respectively, in the SRL INC. (Tokyo, Japan). The working ranges for the FDP and TAT assay were ≤96 mg/dL and ≤120 ng/mL, respectively. Cases showing the upper limit of measurement were not retested, and these values were used in the analysis. We used data from the first measurement after delivery for each patient for analysis. The data used in this study were fully anonymized.

### 2.3. Validation of Boundary Criterion for Predicting Hematuria and Comparison of Coagulation—Fibrinolytic Activation in Each Group

To validate the boundary criterion for predicting hematuria, we used the following formula to calculate the value for severe PA cases with fibrinogen < 170 mg/dL: if the calculated value of the formula “FDP–fibrinogen/3–60 (mg/dL)” was positive, the case was diagnosed as hematuria; otherwise, non-hematuria was observed [12]. We then examined whether the results obtained using the formula were consistent with the clinical hematuria complications in each case.

Additionally, to elucidate the characteristics of the coagulation–fibrinolytic activation in the PPH with hematuria, PPH without hematuria, and PA without hematuria cases, we compared fibrinogen, FDP, TAT, and PIC levels among these groups.

### 2.4. Data Analysis

All anonymized data from each center were analyzed. Continuous variables are reported with the median and range. Categorical variables are reported as counts with percentages. For comparison among the three groups, we used the Kruskal–Wallis test and the χ^2^ test for the continuous and categorical variables, respectively. We used the Mann–Whitney test for the continuous variables for comparison between the two groups and GraphPad Prism 8.4.3 (GraphPad Software, San Diego, CA, USA) for all analyses, with *p* < 0.05 set as the level of significance.

This study was approved by the NHO Central Research Ethics Committee (R1-1009002, 7 February 2020) and was conducted with reference to the STROBE statement [19].

## 3. Results

**Figure 1:**
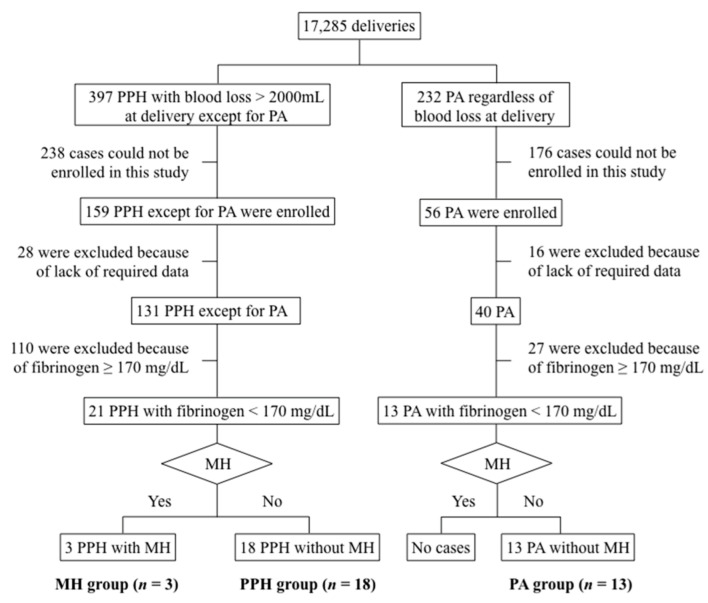
A flowchart of the study participants. Data were collected for women with >2000 mL of blood loss within 24 h of delivery and women with clinically diagnosed PA regardless of blood loss. The boundary criterion for predicting hematuria was developed using severe cases of PPH with fibrinogen < 170 mg/dL [12]. Therefore, this study used PA cases with fibrinogen < 170 mg/dL to validate the boundary criterion. MH, macroscopic hematuria; PA, placental abruption; PPH, postpartum hemorrhage.

Figure 1 shows a flowchart of the study participants. During the study period, 159 cases of PPH with blood loss > 2000 mL at delivery, except for PA, and 56 cases of PA regardless of blood loss at delivery were included among 17,285 deliveries at all participating centers. Of these women, 131 with PPH and 40 with PA fulfilled the inclusion criteria, and 21 PPH and 13 PA with fibrinogen < 170 mg/dL were obtained for this study’s final sample. Of 21 PPHs, 3 were MH (the MH group), and 18 were not (the PPH group). All 13 PAs were without MH (the PA group).

Table 1 shows the clinical characteristics in the three groups. Of 13 cases in the PA group, 12 underwent an emergency C/S because of a deterioration in the fetal heart rate when monitoring; thus, both the Apgar score (median, 1 point) and umbilical arterial pH (median, 7.045) in the PA group were low. Two of three women in the MH group had complicated mild gestational hypertension and mild pre-eclampsia, respectively, and underwent an emergency C/S at 37 and 38 weeks of gestation, respectively, because of elevated blood pressure. Another one underwent an elective C/S at 32 weeks of gestation because of fetal indication and was diagnosed with an adherent placenta intraoperatively.

**Figure 2:**
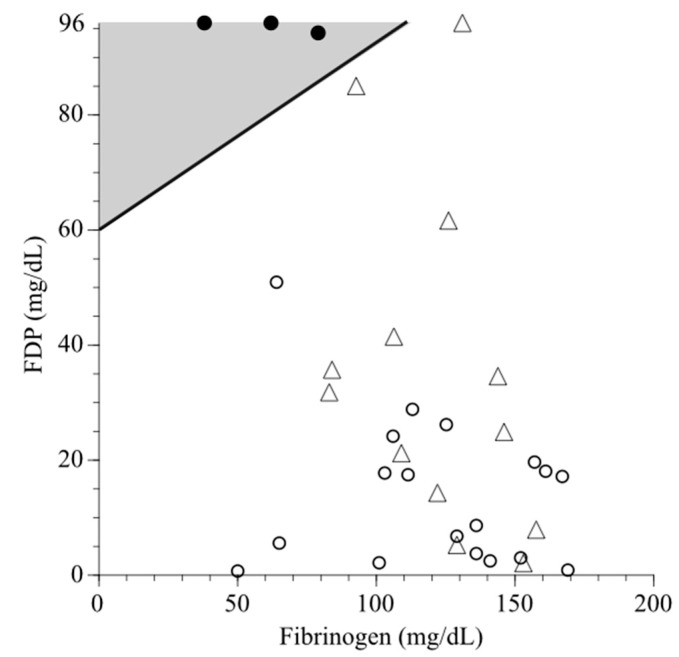
Scatterplots of the cases in the MH (●), PPH (○), and PA groups (△) on the fibrinogen–FDP plane, respectively. The gray area indicates hematuria. The boundary was a straight line connecting (0, 60) and (108, 96) on the fibrinogen–FDP plane. A positive FDP–fibrinogen/3–60 (mg/dL) value indicates hematuria; otherwise, non-hematuria is indicated [12]. The calculated values in 13 cases in the PA group (△) were negative, and all cases were under the boundary. All three cases in the MH group (●) that we considered as clinical DIC were over the boundary. See Figure 1 for the details of each group. FDP, fibrin/fibrinogen degradation product; MH, macroscopic hematuria; PA, placental abruption; PPH, postpartum hemorrhage.

Figure 2 shows scatterplots of the cases in the three groups on the fibrinogen–FDP plane. The calculated values using the boundary criterion formula for 13 cases in the PA group ranged from −108.91 to −5.87; all were negative, and all cases were diagnosed as non-hematuria. Therefore, when the (fibrinogen and FDP) coordinates of each case in the PA group (△) were plotted on the fibrinogen–FDP plane (on which the boundary for predicting hematuria is shown as a solid line), all cases were under the boundary.

Table 2 shows a comparison of the laboratory tests results and clinical data among the three groups. For the coagulation–fibrinolytic tests, there were significant differences for fibrinogen, FDP, D-dimer, and PIC among the three groups, but TAT levels did not differ. FDP and PIC levels in the PA group were higher (*p* < 0.05) than in the PPH group, but fibrinogen and TAT levels did not differ between the two groups. Blood loss at the initial blood sampling in the PA group was smaller (*p* < 0.01) than in the PPH group.

**Figure 3:**
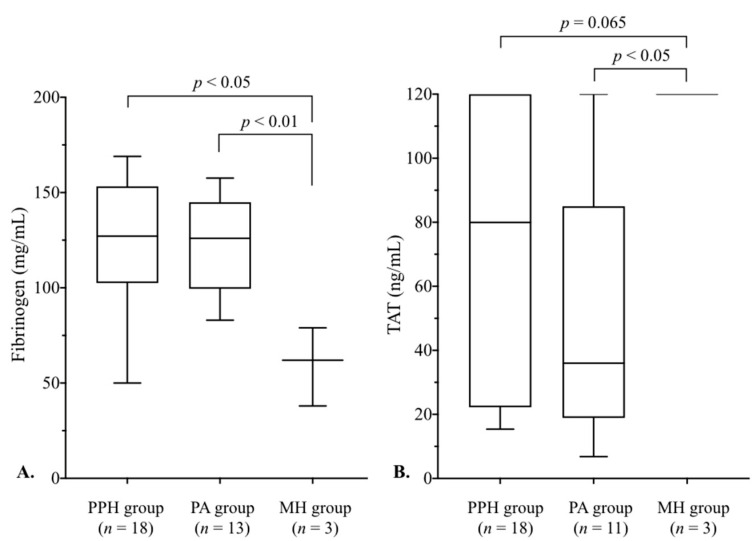
A comparison of fibrinogen and TAT levels among the three groups. (**A**) Fibrinogen levels in the MH group were lower than in the PPH (*p* < 0.05) and PA (*p* < 0.01) groups. (**B**) TAT levels in the MH group were higher (*p* < 0.05) than in the PA group and tended to be higher (*p* = 0.065) than in the PPH group. The boxes represent the 25th and 75th percentiles, and the whiskers are the maximum and minimum values. See Figure 1 for the details of each group. MH, macroscopic hematuria; PA, placental abruption; PPH, postpartum hemorrhage; TAT, thrombin–antithrombin complex.

Figure 3 shows a comparison of fibrinogen and TAT levels among the three groups. Fibrinogen levels in the MH group were lower than in the PPH (*p* < 0.05) and PA (*p* < 0.01) groups (Figure 3A). TAT levels did not differ (*p* = 0.054) among the three groups (Table 2); however, TAT levels in the MH group, all at the upper limit of measurement, were higher (*p* < 0.05) than in the PA group and tended to be higher (*p* = 0.065) than in the PPH group. Fibrinogen and TAT levels did not differ between the PA and PPH groups.

**Figure 4:**
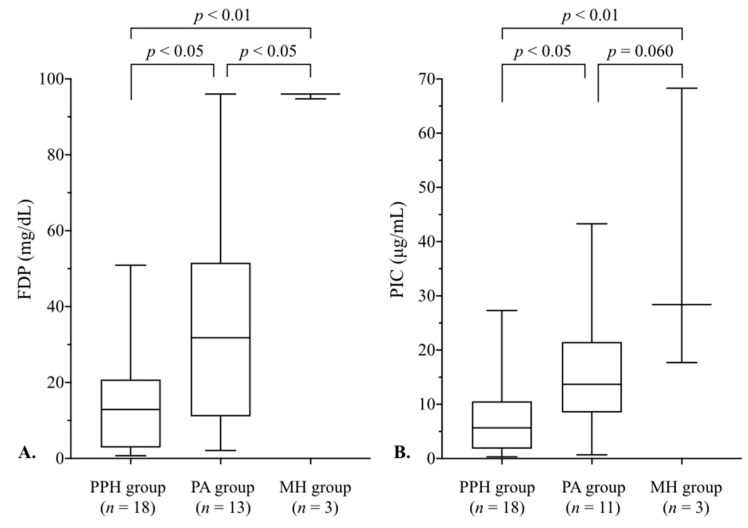
A comparison of FDP and PIC levels among the three groups. (**A**) FDP levels in the MH group were higher than in the PPH (*p* < 0.01) and PA (*p* < 0.05) groups. (**B**) PIC levels in the MH group were higher (*p* < 0.01) than in the PPH group and tended to be higher (*p* = 0.060) than in the PA group. The boxes represent the 25th and 75th percentiles, and the whiskers are the maximum and minimum values. See Figure 1 for the details of each group. FDP, fibrin/fibrinogen degradation product; MH, macroscopic hematuria; PA, placental abruption; PIC, plasmin-α_2_–plasmin inhibitor complex; PPH, postpartum hemorrhage.

Figure 4 shows a comparison of FDP and PIC levels among the three groups. FDP levels in the MH group were higher than in the PPH (*p* < 0.01) and PA (*p* < 0.05) groups (Figure 4A). PIC levels in the MH group were also higher than in the PPH group (*p* < 0.01) and tended to be higher (*p* = 0.060) than in the PA group (Figure 4B). Both FDP and PIC levels in the PA group were higher (*p* < 0.05) than in the PPH group.

## 4. Discussion

This study demonstrated that the boundary criterion formula for predicting hematuria—FDP–fibrinogen/3–60 (mg/dL) [12]—accurately diagnosed all cases of severe PA without hematuria as non-hematuria cases. The types of coagulopathies during delivery were classified as dilutional or consumptive, with the latter further divided into local consumptive and DIC [16]. Most consumptive coagulopathy cases are associated with PPH results from the local consumption of fibrinogen, forming hemostatic clots in the placental vascular bed or myometrial vessels (e.g., PA, adherent placenta, or atony) [16,20]. The cases in the PPH and PA groups below the boundary in Figure 2 corresponded to this condition, and coagulation–fibrinolytic system activation was localized to the uterus. DIC onset in obstetrics is not only related to the influx of massive tissue factors from damaged maternal decidua [21,22] but also to the involvement of the innate immune system [10,21], resulting in the systemic activation of coagulation and fibrinolysis. Cases of clinical DIC in the MH group (Figure 2) corresponded to this condition, and it is thought that in this group, fibrinogen decreased rapidly and FDP increased rapidly due to enhanced coagulation and fibrinolysis activation [4,9].

In the MH group, TAT levels were all at the upper limit of measurement, and PIC levels (median, 28.4 μg/mL) were also high compared to the other two groups (Figure 3 and Figure 4). Additionally, PIC levels were high, similar to those in a recent report [23] that showed elevated PIC levels in clinical AFE (median, 60 μg/mL) and PA (median, 10 μg/mL) cases with DIC. These results in the MH group illustrated enhanced coagulation–fibrinolytic activation, the main pathophysiology of obstetrical DIC [4]. Moreover, FDP in the MH group was markedly elevated (Figure 4). FDP is a degradation product of fibrinogen and fibrin derived by plasmin; thus, high FDP levels in the MH group reflected the enhanced fibrinogenolysis [24], resulting in a marked decrease in fibrinogen levels (Figure 3). Considering these laboratory tests results, cases with hematuria could be assumed to have clinical DIC, but further confirmation via additional data is needed.

It is becoming clearer that damage-associated molecular patterns (DAMPs) from host cells damaged by infection and trauma play a crucial role in the onset of DIC in sepsis [25] and trauma [7]. Innate immune cells activated by DAMPs release inflammatory cytokines, which activate the coagulation system systemically [7,26], leading to DIC onset. In AFE, it is believed that some amniotic fluid components that function as DAMPs [27] enter the maternal circulation and cause DIC [28]. Leong et al. reported that the presence of amniotic fluid components within the myometrial vessels obtained during C/S is a common finding [29]; this suggests that amniotic fluid enters the maternal circulation. Additionally, in vitro data have shown that even a small amount of amniotic fluid causes maternal whole blood coagulation [30,31]. Considering the above, we suppose that in the MH cases delivered by C/S, the influx of amniotic fluid into the maternal circulation following C/S may have played some role in the onset of DIC; however, the exact mechanism is unclear.

Blood loss at the initial blood sampling in the PA group (median, 1594 mL) was smaller (*p* < 0.01) than that in the PPH group (2598 mL), but fibrinogen levels were similarly low in both groups (Figure 3A). Focusing on the coagulation and fibrinolysis system in the PA group, the TAT level did not differ from that in the PPH group (Figure 3B), but the fibrinolysis markers’ FDP and PIC levels were both significantly higher than in the PPH group (Figure 4). Unlike PPH, e.g., atony or a birth canal injury, the disruption of immune processes in the decidua plays an important role in PA development, leading to a maternal immune response with the release of cytokines [22,32]. Considering that some severe PA cases develop DIC [10,16], PA may cause hyperfibrinolysis even if it does not progress to DIC, contributing to a low fibrinogen level despite minimal blood loss. This study did not allow for a comparison of coagulation–fibrinolytic activation with normal deliveries, but it is meaningful that in severe obstetrical conditions, there were differences in its activation depending on the pathophysiology.

This study had several limitations. First, there was a low number (three) of clinical DIC cases. A study of 151,678 deliveries in a tertiary center showed a lower incidence of DIC, at 0.032% [6], while a rough calculation of the incidence rate for this study was also low, at 0.017% (3/17,285), indicating that this is a rare condition. There also was an upper limit to the TAT and FDP measurements, and the severity of coagulation–fibrinolytic activation in the MH group may have been underestimated. We also were unable to examine the detailed mechanism of coagulation–fibrinolytic system activation, as we did not measure proinflammatory cytokines that activate the coagulation system [7], and factors that affect the fibrinolysis system, such as tissue plasminogen activators [23].

## 5. Conclusions

This study of patients with severe PA without hematuria confirmed the accuracy of the hematuria prediction formula, “FDP–fibrinogen/3–60 (mg/dL)”, consisting of fibrinogen and FDP, which can be rapidly measured at many facilities. Additionally, the results of coagulation–fibrinolysis tests including TAT and PIC suggested that cases with hematuria could be considered clinical DIC. However, as there were only three cases with hematuria, further cases need to be studied. If the validity of this criterion is confirmed in many hematuria cases in the future, it may become a useful criterion for diagnosing clinical DIC during delivery.

## Figures and Tables

**Table 1:** The clinical characteristics of participants in the three groups. Values are presented as the median (range) or counts (%).

	PPH Group (*n* = 18) (PPH Without MH)	PA Group (*n* = 13) (PA Without MH)	MH Group (*n* = 3) (PPH with MH)	*p*-Value
Maternal characteristics				
Age (years)	37 (28–45)	32 (22–45)	32 (28–36)	0.20
Nulliparity (%)	10 (56)	3 (23)	2 (67)	0.14
Body mass index at delivery (kg/m^2^)	26.1 (20.7–28.1)	27.6 (22.6–38.6)	25.6 (20.6–28.6)	0.12
Gestational age at delivery (weeks)	37.5 (28–41)	35 (22–39)	37 (32–38)	<0.05
HDP complicated				
Gestational hypertension (%)	1 (6)	1 (8)	1 (33)	0.29
Pre-eclampsia (%)	6 (33)	6 (46)	1 (33)	0.76
Neonatal characteristics				
Apgar score at 1 min	8 (1–10)	1 (0–8)	8 (1–8)	<0.001
Umbilical arterial pH	7.292 (7.051–7.351)	7.045 (6.563–7.282)	7.228 (7.225–7.296)	<0.001
Mode of delivery				
Cesarean section (%)	9 (50)	12 (92)	3 (100)	<0.05
Instrumental vaginal (%)	2 (11)	0 (0)	0 (0)	0.39
Multifetal pregnancy (%)	4 (22)	0 (0)	1 (33)	0.14
Maternal transport (%)	6 (33)	5 (38)	0 (0)	0.44
Causes of bleeding				
Uterine atony (%)	8 (44)	0 (0)	0 (0)	–
Placenta previa (%)	2 (33)	0 (0)	0 (0)	–
Adherent placenta (%)	4 (33)	0 (0)	1 (33)	–
Surgical trauma (%)	3 (17)	0 (0)	2 (67)	–
Others (%)	1 (6)	0 (0)	0 (0)	–
PA (%)	0 (0)	13 (100)	0 (0)	–

HDP, hypertensive disorder of pregnancy; MH, macroscopic hematuria; PA, placental abruption; PPH, postpartum hemorrhage.

**Table 2:** A comparison of laboratory tests and clinical data among the three groups. Values are presented as the median (range) or counts (%).

	PPH Group (*n* = 18) (PPH Without MH)	PA Group (*n* = 13) (PA Without MH)	MH Group (*n* = 3) (PPH with MH)	*p*-Value
Hemoglobin (g/dL)	6.75 (5–10.2)	8.3 (4.9–11.3)	7.5 (4.5–10.1)	<0.05
Platelet counts (×1000/μL)	88 (39–147)	120 (45–204)	96 (76–144)	0.31
Prothrombin time–INR	1.11 (0.96–10)	1.1 (1.02–1.44)	1.53 (1.46–1.84)	<0.05
Fibrinogen (mg/dL)	127.1 (50–169)	126 (83–158)	62 (38–79)	<0.05
FDP (mg/dL)	12.9 (0.72–50.93)	31.8 (2.09–96)	96 (94.73–96)	<0.01
D-dimer (mg/dL)	2.875 (0.3–21.9)	3.7 (0.65–26.69)	42.55 (13.54–49.1)	<0.05
TAT (ng/mL)	79.95 (15.4–120)	36 (6.8–120) ^a^	120 (120–120)	0.054
PIC (μg/mL)	5.65 (0.3–27.3)	13.7 (0.7–43.3) ^a^	28.4 (17.7–68.3)	<0.01
Time between delivery and initial blood sampling (h)	2.83 (1–14)	2 (0.56–8)	1.5 (1.3–4)	0.30
Blood loss at initial blood sampling (mL)	2598.5 (900–4000)	1594 (404–4294)	2215 (1300–2215)	<0.05
Total blood loss (mL)	3148 (2015–5294)	1856 (475–4294)	2950 (2700–3801)	<0.001
Volume of RCC at initial blood sampling (mL)	0 (0–1120)	0 (0–2240)	0 (0–0)	0.44
Total volume of RCC (mL)	840 (0–3360)	560 (0–2520)	1120 (840–1960)	0.18
Volume of FFP at initial blood sampling (mL)	240 (0–1200)	480 (0–3600)	0 (0–0)	0.42
Total volume of FFP (mL)	720 (0–4800)	960 (0–4080)	960 (720–1920)	0.72

FDP, fibrin/fibrinogen degradation product; FFP, fresh frozen plasma; INR, international normalized ratio; MH, macroscopic hematuria; PA, placental abruption; PIC, plasmin-α_2_–plasmin inhibitor complex; PPH, postpartum hemorrhage; RCC, red cell concentrate; TAT, thrombin–antithrombin complex. ^a^ Data are available for 11 patients.

## Data Availability

The datasets generated during this study are available from the corresponding author upon reasonable request.

## Abbreviations

The following abbreviations are used in this manuscript:

AFE	Amniotic fluid embolism
C/S	Cesarean section
DAMPs	Damage-associated molecular patterns
DIC	Disseminated intravascular coagulation
FDP	Fibrin/fibrinogen degradation product
FFP	Fresh frozen plasma
HDP	Hypertensive disorder of pregnancy
INR	International normalized ratio
ISTH	International Society of Thrombosis and Haemostasis
MH	Macroscopic hematuria
NHO	National Hospital Organization
PA	Placental abruption
PIC	Plasmin-α_2_–plasmin inhibitor complex
PPH	Postpartum hemorrhage
RCC	Red cell concentrate
TAT	Thrombin–antithrombin complex
